# Polyproline as a Minimal Antifreeze Protein Mimic That Enhances the Cryopreservation of Cell Monolayers

**DOI:** 10.1002/anie.201706703

**Published:** 2017-11-22

**Authors:** Ben Graham, Trisha L. Bailey, Joseph R. J. Healey, Moreno Marcellini, Sylvain Deville, Matthew I. Gibson

**Affiliations:** ^1^ Department of Chemistry University of Warwick Gibbet Hill Road Coventry CV47 AL UK; ^2^ Warwick Medical School University of Warwick Coventry CV4 7AL UK; ^3^ Ceramics Synthesis and Functionalization Lab UMR3080 CNRS/Saint-Gobain 550 Avenue Alphonse Jauffret 84306 Cavaillon France

**Keywords:** biomaterials, cryopreservation, ice recrystallization inhibitors, monolayers, polymers

## Abstract

Tissue engineering, gene therapy, drug screening, and emerging regenerative medicine therapies are fundamentally reliant on high‐quality adherent cell culture, but current methods to cryopreserve cells in this format can give low cell yields and require large volumes of solvent “antifreezes”. Herein, we report polyproline as a minimum (bio)synthetic mimic of antifreeze proteins that is accessible by solution, solid‐phase, and recombinant methods. We demonstrate that polyproline has ice recrystallisation inhibition activity linked to its amphipathic helix and that it enhances the DMSO cryopreservation of adherent cell lines. Polyproline may be a versatile additive in the emerging field of macromolecular cryoprotectants.

Tissue engineering, gene therapy, therapeutic protein production, and transplantation rely on the successful storage and transport of donor cells.^1^ For example, in the production of therapeutic proteins, a specific cell line must be developed for each protein.^2^ Given that any in vitro culture will undergo phenotypic and genotypic changes when propagated for long periods of time, it is neither possible nor practical to maintain a continuous culture of cells.^3^ The only solution to this is the cryopreservation of cells using significant volumes of cryoprotectants, such as DMSO (dimethyl sulfoxide), which are intrinsically toxic.^4^ The repeated use of DMSO has an impact on the epigenetic profile of cells, specifically the alteration of DNA methylation profiles, which results in phenotypic changes.^5^, ^6^ There is a real need for robust methods to cryopreserve cells in monolayer (adhered to tissue culture scaffolds) format to provide phenotypically identical cells for assays, obviating the need for replating between freeze–thaw cycles. Formulations containing 5–10 % DMSO reduce cryoinjury by moderating the increase in solute concentration during freezing^7^, ^8^, ^9^ but for adhered embryonic stem cells, their use results in just 5 % cell recovery.^10^, ^11^ A key contributor to cell death during cryopreservation is ice recrystallisation (growth) and additives that can inhibit recrystallisation have the potential to redefine cell storage and hence biomedicine.

Antifreeze (glyco)proteins (AF(G)Ps) are potent ice recrystallisation inhibitors (IRIs), but are unsuitable for cryopreservation applications owing to their potential toxicity/immunogenicity and their secondary effect of dynamic ice shaping (DIS), which leads to needle‐like ice crystals that pierce cell membranes.^12^ Synthetic polymers that are potent IRIs have emerged as new tools for controlling ice growth.^13^ The most studied one is poly(vinyl alcohol) (PVA), which can inhibit ice growth at concentrations below 0.1 mg mL^−1^ and enhances the cryopreservation of cells in suspension.^14^, ^15^, ^16^ It is hypothesized that the activity of PVA is related to its regularly spaced hydroxyl groups.^17^ Matsumura and Hyon have developed polyampholytes^18^ that are cryoprotective but have moderate IRI activity.^19^, ^20^ Wang and co‐workers have demonstrated the significant IRI activity of graphene oxide.^21^ Ben and co‐workers have developed low‐molecular‐weight surfactants that also inhibit ice growth.^22^ A major setback is that the above synthetic IRIs are neither biodegradable nor bioresorbable and have not been applied to the significant challenge of cell monolayer storage.

There are no crystal structures for AFGPs but solution‐state NMR and circular dichroism (CD) spectroscopy suggest a polyproline II (PP II)‐type helix.^23^ Polyproline is unique amongst the canonical amino acids in that it has no amide N−H, meaning that it cannot form intramolecular hydrogen bonds. Therefore, it is water‐soluble and quite hydrophobic at the same time, as is the case for AFP I, which contains 70 % alanine (a hydrophobic amino acid). We thus hypothesised that polyproline could be a minimal AF(G)P mimic owing to its amphiphilicity.^24^ Homopolypeptides are appealing targets compared to vinyl polymers as they can be prepared by solid‐phase synthesis,^25^ solution‐phase polymerisation,^26^ or recombinant methods,^27^ proving vast (bio)synthetic space.

Herein, we introduce polyproline as a minimum (bio)synthetic antifreeze protein mimic. We demonstrate that polyproline has ice recrystallisation inhibition activity, which is linked to its amphipathic PP II helix structure. Polyproline was found to improve the post‐cryopreservation recovery of cell monolayers compared to DMSO alone, demonstrating a new macromolecular approach for the storage of complex cells to enable next‐generation therapies.

l‐, d‐, and (racemic) d/l‐polyproline were synthesised by condensation polymerisation using 1‐ethyl‐3‐(3‐dimethylaminopropyl) carbodiimide (EDC, Scheme 1), alongside several commercial samples. Following dialysis, the polymers were characterised by size exclusion chromatography (SEC; Table 1). The polymers were less disperse than expected owing to fractionation during dialysis.

**Scheme 1:**
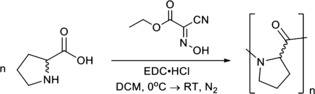
Condensation polymerisation of proline. The materials were used in stereopure form but both the l‐ and d‐isomers were used, hence no stereocentres are shown.

**Table 1:** Polyproline characterisation.

	*M*_n_ [g mol^−1^]	*Đ* ^SEC [a]^	DP	Secondary structure
PPro_11_	1300^[a]^	1.03	11	PP II
PPro_15_	1700^[a]^	2.12	15	
PPro_19_	2100^[a]^	1.50	19	
P(d‐Pro)_15_	1700^[a]^	1.01	15	enantiomeric PP II
P(d/l‐Pro)_21_	2400^[a]^	1.01	21	random coil
PPro_10‐100_	1–10 000^[b]^	–	10–100	PP II^[e]^
PPro_10_	900^[c]^	^[d]^	10	PP II^[e]^
PPro_10‐25_	1–3000	1.01–1.03	10–25	PP II^[e]^
PPro_20_	2000^[c]^	^[d]^	20	PP II^[e]^

[a] Determined by SEC. [b] Value from supplier. [c] Determined by mass spectrometry. [d] Single species. [e] From Ref. 28, 29, 30.

CD spectroscopy confirmed that PPro_15_ adopted a PP II helix (Figure 1 A; see also the Supporting Information, Figure S1)^31^ with characteristic signals present at 207 and 228 nm, whilst a random coil would exhibit slight peak shifting, with signals absent in the 220 nm region.^32^ P(d‐Pro)_15_ gave the mirror spectrum whilst the d/l racemic mixture showed no secondary structure. This series of peptides were subsequently tested for IRI activity using a splat assay.^33^ This involved seeding a large number of small ice crystals, which were annealed for 30 min at −8 °C before being photographed. The average crystal size was measured relative to a PBS control, with smaller values indicating more IRI activity (Figure 1 B, C).

**Figure 1:**
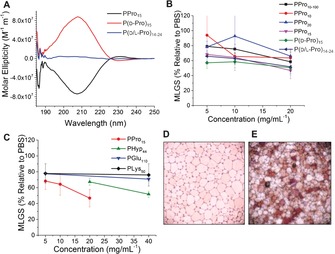
A) Circular dichroism spectra. B) IRI activity of the polyproline series. C) IRI activity compared to other homopolypeptides. D) Cryomicrograph of a PBS negative control. E) Cryomicrograph of 20 mg mL^−1^ polyproline. Photographs taken after 30 min at −8 °C. Error bars represent ± standard deviation from a minimum of three replicates. Images shown are 1.2 mm across. MLGS=mean largest grain size.

All polyproline variants were found to display dose‐dependent activity but weak molecular‐weight dependence in the range tested (Figure 1 B). The shortest peptide (PPro_10_) lost activity below 10 mg mL^−1^, but the longer ones retained activity at 5 mg mL^−1^. The magnitude of activity was significantly smaller than for AF(G)Ps, which function at concentrations as low as 0.14 μg mL^−1^,^34^ but comparable to that of polyampholytes.^19^, ^20^ Knight and co‐workers have observed that poly(hydroxyproline) has IRI activity, which was assumed to be due to the regularly spaced hydroxyl groups along the backbone.^35^ However, the observations made here suggest that the PP II helix, rather than (or in addition to) the hydroxyl groups, gives rise to the observed activity. Figure 1 C compares the IRI activity of poly(hydroxyproline) with those of PPro_15_ and two α‐helical poly(amino acid)s.^36^ Polylysine (PLys_50_) and poly(glutamic acid) (PGlu_110_) showed no IRI activity. PPro_15_ was found to be more active than poly(hydroxyproline) of higher molecular weight. This finding confirmed that hydroxyl groups are not essential for activity in IRI‐active compounds. P(d‐Pro_15_) and P(d/l‐Pro_21_) had statistically identical activity to PPro_15_, suggesting that local rather than long‐range order is crucial for activity.

We hypothesise that IRI activity requires segregated hydrophilic and hydrophobic domains (amphipathy).^22^, ^24^, ^37^ PPro_10_ was compared to a non‐glycosylated type I sculpin AFP^38^ and also to PGlu_10_ by mapping their hydrophobic/hydrophilic domains (Figure 2). The type I sculpin AFP (Figure 2 A) possesses “patches” of hydrophobic/hydrophilic groups. PPro_10_ (Figure 2 B) also possesses this facial amphiphilicity. In comparison, PGlu_10_ (no IRI activity) has charged groups around the core of the helix, which prevents the presentation of hydrophobic domains. This agrees with our previous study on nisin A, which has IRI activity associated with segregated domains,^37^ and also the results obtained with amphiphiles developed by Ben et al., which only function below the critical micelle concentration.^22^

**Figure 2:**
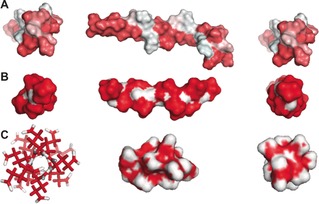
Hydrophobic surface mapping of A) recombinant type I sculpin AFP, B) PPro_10_, and C) PGlu_10_, showing charged hydrophilic surfaces. Hydrophobic regions (red), hydrophilic regions (white).

Aside from IRI activity, AF(G)Ps display unwanted ice shaping, which promotes the formation of needle‐like ice crystals, which damage cell membranes.^12^ Cryo‐confocal microcapillary microscopy has emerged as a tool for monitoring ice crystal shaping,^39^ and was also employed here (Figure 3). A non‐IRI‐active dye, sulforhodamine B, provided contrast against the ice (which appears dark). A control using pure PBS showed no shaping whilst zirconium acetate (ZrAc), which is a strong ice shaper, produced hexagonal crystals.^39^ PPro_19_ did not induce shaping, supporting the concept that polyproline inhibits ice crystal growth without inhibiting the formation of a specific plane of ice; however, as these are relatively weak IRIs, the concentrations needed for ice shaping would be very high.

**Figure 3:**
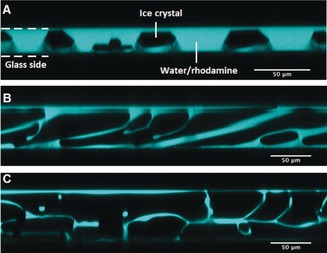
Cross‐section of ice crystals perpendicular to the temperature gradient: A) ZrAc (positive control), B) PPro_19_, C) PBS (negative control). The ice crystals expel the dye while growing, appearing in black, while the remaining liquid fluoresces.

To explore polyproline as a macromolecular cryopreservative, A549 cells were employed as a prototypical adherent cell line.^40^ The protective osmolyte proline (which has no IRI activity; see the Supporting Information) was used as a secondary cryoprotectant. A549 cells were incubated with 200 mm (23 mg mL^−1^) proline (blue bars; Figure 4) or medium alone (red bars; Figure 4) for 24 h. The medium was then removed and replaced with a medium containing 10 % DMSO with varying concentrations of PPro_11_ (1250 g mol^−1^, *Đ*=1.03). After 10 min exposure to this solution, all excess solvent was removed, and the cells were subjected to controlled‐rate freezing at 1 °C min^−1^ to −80 °C. Following storage at −80 °C, the cells were thawed by addition of warm medium (37 °C), and the total number of viable cells was determined by trypan blue staining 24 h after thawing. Figure 4 shows that the use of DMSO alone led to 27 % cell recovery. Addition of polyproline alone to 10 % DMSO failed to give any additional protection. However, for cells that had been preconditioned with 200 mm proline for 24 h before treatment with 10 mg mL^−1^ PPro_11_/10 % DMSO, the cell recovery doubled to 53 %. Increasing the concentration of polyproline beyond 10 mg mL^−1^ did not increase recovery further, suggesting that the additive benefits plateau at 10 mg mL^−1^.^14^ It should be highlighted that the cell viability assays measure intact cells, and that detailed functional analysis will be needed in the future for demonstration of complex function. For comparison with other macromolecular cryopreservatives, Matsumura and co‐workers have reported poly(ampholyte)‐enhanced monolayer storage using vitrification solutions, giving near‐quantitative cell recovery.^41^ However, this required very high DMSO concentrations of 6.5 m (>500 mg mL^−1^) plus 10 wt % (ca. 100 mg mL^−1^) of the polymer, and there was a reduction in the post‐thaw proliferation rate associated with the large solvent volumes, which may limit practical applications. In our PPro system introduced here, the total recovery levels were less, but far lower concentrations of DMSO were employed (10 wt %/ca. 100 mg mL^−1^), and the total exposure time to this potentially toxic component was only 10 min. To critically compare PPro, another batch (PPro_10–25_) was synthesised and tested for cytotoxicity and heamocompatibility. A549 monolayers were exposed to PPro for 24 h, and the cell viability was assessed (see the Supporting Information). This extended exposure period led to a reduction in alamar blue to 60 % for 5 mg mL^−1^ PPro, suggesting some cytotoxicity if exposed to elevated concentrations for long periods of time. It is important to note that in this cryopreservation procedure, PPro is only in contact with the cells for 10 min before the excess is removed and the cells are frozen. Red blood cell heamolysis experiments (see the Supporting Information) showed this was not due to any inherent membrane activity of the (amphipathic) PPro.

**Figure 4:**
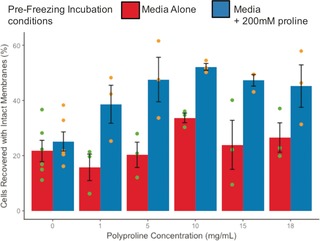
A549 cryopreservation. Cell recovery determined by trypan blue assays. Cells were first incubated either in the medium alone or with 200 mm proline for 24 h. They were subsequently cryopreserved by addition of 10 % DMSO with the indicated PPro_11_ concentration. Error bars ± S.E.M. from *n*=3 with two nested replicates. # *P*<0.05 compared to 10 % DMSO treatment; * *P*<0.05 compared to 200 mm proline exposure with 10 % DMSO treatment.

In summary, we have demonstrated that polyproline is a potent additive for cell‐monolayer cryopreservation when appropriate freezing conditions are employed. Polyproline has moderate ice recrystallisation inhibition activity, which was hypothesised to be due to its “patchy” amphipathic structure associated with its PP II helix. Addition of polyproline to adherent cell cultures led to an increase from 20 % to >50 % in total cell recovery post‐cryopreservation, which is significantly better than for the use of DMSO alone. This increase in recovery is thought to be associated with the inhibition of ice recrystallisation. Short exposure times of just 10 min to the polyproline/DMSO solution, followed by removal of the excess solvent, reduced the cytotoxicity associated with long‐term (24 h) exposure to elevated levels of polyproline. The minimal solvent exposure times may give benefits in downstream processing and biomedical applications compared to current high‐solvent‐concentration methods using vitrification. Polyproline is appealing compared to other macromolecular cryoprotectants as it only comprises native amino acids and can be obtained by chemical and biochemical methods.

## Conflict of interest

The authors declare no conflict of interest.

## Supporting information

As a service to our authors and readers, this journal provides supporting information supplied by the authors. Such materials are peer reviewed and may be re‐organized for online delivery, but are not copy‐edited or typeset. Technical support issues arising from supporting information (other than missing files) should be addressed to the authors.

Supplementary
