# Supplementary material for: Polyproline as a Minimal Antifreeze Protein Mimic That Enhances the Cryopreservation of Cell Monolayers
Source: Angew Chem Int Ed Engl. 2017 Nov 22;56(50):15941–4. doi: 10.1002/anie.201706703 (PMC5722203; doi:10.1002/anie.201706703)
Supplement: Supplementary file 1 — Supplementary [file ANIE-56-15941-s001.pdf]

Angewandte Chemie
Eine Zeitschrift der Gesellschaft Deutscher Chemiker

Supporting Information

Polyproline as a Minimal Antifreeze Protein Mimic That Enhances the Cryopreservation of Cell Monolayers

*Ben Graham, Trisha L. Bailey, Joseph R. J. Healey, Moreno Marcellini, Sylvain Deville, and Matthew I. Gibson**

anie_201706703_sm_miscellaneous_information.pdf

Materials

L and D-proline, poly-L-proline mol wt 1,000-10,000 ($PPro_{10-100}$), ethyl (hydroxyimino) cyanoacetate (OxymaPure^{TM}), *N*-(3-dimethylaminopropyl)-*N'*-ethylcarbodiimide hydrochloride (EDCI), dichloromethane (DCM), phosphate-buffered saline preformulated tablets, and hydrochloric acid (37%) were purchased from Sigma Aldrich Co Ltd (Gillingham, UK) and used without further purification. Dialysis Membrane Spectra/Por 7 Flexible 38mm FWT 1000 MWCO 4.6 mL/cm was purchased from Fischer Scientific (Loughborough, UK) and used directly. Phosphate-buffered saline (PBS) solution was prepared using preformulated tablets in 200 mL of Milli-Q water (>18.2 Ω mean resistivity) to give [NaCl] = 0.138 M, [KCl] = 0.0027 M, and pH 7.4. $PPro_{10}$ and $PPro_{20}$ (>90%) were purchased bespoke from Peptide Protein Research Ltd (Fareham, UK) and were used without further purification. $PPro_{10}$: m/z (ESI) 988.0 (100%, -1); $PPro_{20}$: m/z (ESI) 491.0 (20%, +4), 654.3 (100%, +3), 981.0 (30%, +2).

Physical and analytical methods

SEC (size exclusion chromatography) was acquired a DMF Agilent 390-LC MDS instrument equipped with differential refractive index (DRI), viscometry (VS), dual angle light scatter (LS) and dual wavelength UV detectors. The system was equipped with 2 x PLgel Mixed D columns (300 x 7.5 mm) and a PLgel 5 µm guard column. The eluent is DMF with 5 mmol NH4BF4 additive. Samples were run at 1 mL/min at 50'C. Poly(methyl methacrylate) standards (Agilent EasyVials) were used for calibration. Analyte samples were filtered through a nylon membrane with 0.22 µm pore size before injection. Respectively, experimental molar mass (Mn,SEC) and dispersity (Đ) values of synthesized polymers were determined by conventional calibration (relative to poly(methyl methacrylate) standards) using Agilent GPC/SEC software. Refractive index recorded.

General procedure for the synthesis of polyproline peptides $PPro_n$

EDCI (0.50 g, 2.60 mmol) was dissolved in dry DCM (20 mL) and stirred at room temperature under a flow of nitrogen for 20 minutes, followed by cooling to 0 ºC. Within 5 minutes of cooling, L-proline (0.30 g, 2.60 mmol, 1 eqv) and OxymaPure™ (0.37 g, 2.60 mmol, 1 eqv) were added together to the reaction mixture, resulting in an instantaneous colour change to yellow. The mixture was stirred on ice under nitrogen for 1 further hour, and then warmed to RT with stirring overnight. The dark yellow solution was condensed *in vacuo*, dissolved in Milli-Q water (10 mL) acidified to pH 3-4 with 3M HCl, and a minimum volume of methanol added until residual solids dissolved. Dialysis (> 1 kDa) for 48 hours was subsequently performed with regular water changes. The resulting solution was freeze dried, yielding an off-white solid. 31.4 mg (10.4%). The DL racemate, $P(DL)Pro_n$, utilised a 1:1 ratio of L- and D-proline (2.60 mmol prolines).

Ice recrystallisation inhibition (splat) assay

Ice recrystallisation inhibition was measured using a modified splat assay. [1] A 10 µL sample of polymer dissolved in PBS buffer (pH 7.4) was dropped 1.40 m onto a chilled glass coverslip, resting on a thin aluminium block placed on dry ice. Upon hitting the coverslip, a wafer with diameter of approximately 10 mm and thickness 10 µm was formed instantaneously. The glass coverslip was transferred onto the Linkam cryostage and held at -8°C under N_2 for 30 minutes. Photographs were obtained using an Olympus CX 41 microscope with a UIS-2 20x/0.45/∞/0-2/FN22 lens and crossed polarizers (Olympus Ltd, Southend-on-Sea, UK), equipped with a Canon DSLR 500D digital camera. Images were taken of the initial wafer (to ensure that a polycrystalline sample had been obtained) and again after 30 minutes. Image processing was conducting using Image J, which is freely available. In brief, five of the largest ice crystals in the field of view were measured and the single largest length in any axis recorded. The average

(mean) of these five measurements was then calculated to find the largest grain dimension along any axis. This was repeated for three individual wafers, and the average (mean) of these three values was calculated to give the mean largest grain size (MLGS). The average value was compared to that of a PBS buffer negative control.

Surface hydrophobicity mapping of proteins

NMR solution phase (AFP Sculpin) and X-Ray crystal structures of proteins and peptides of interest were acquired from the Protein Data Bank and other publically accessible sources, or computationally modelled in-house ($PPro_{10}$ and $PGlu_{10}$). Structures were rendered in PyMOL (Schrödinger LLC, Cambridge, MA), which is freely available for educational use, and surfaces on the structures were displayed. An open source script "color_h" was used to colour the protein surface according to the Eisenberg hydrophobicity scale of its constituent amino acids, from red (hydrophobic) to white (hydrophilic). For the homo-polypeptides where scaling is not possible, aliphatic hydrogen and carbon were defined as hydrophobic whilst oxygen, hydrogen and nitrogen as hydrophilic, utilising the same colour scheme. Due to the lack of hydrogen bond donors in a $PPro_{10}$ PPII helix, this was considered representative.

Cell culture

Human Caucasian lung carcinoma cells (A549) were obtained from the European Collection of Authenticated Cell Cultures (Salisbury, UK) and grown in 175 cm^2 cell culture Nunc flasks (Corning Incorporated, Corning, NY). Standard cell culture medium was composed of Ham's F-12K (Kaighn's) Medium (F-12K) (Gibco, Paisley, UK) supplemented with 10% USA-origin foetal bovine serum (FBS) purchased from Sigma Aldrich Co Ltd (Gillingham, UK), 100 units/mL penicillin, 100 µg/mL streptomycin, and 250 ng/mL amphotericin B (PSA) (HyClone, Cramlington, UK). A549 cells were maintained in a humidified atmosphere of 5% CO_2 and 95% air at 37 °C and the culture medium was renewed every 3–4 days. The cells were

subcultured every 7 days or before reaching 90% confluency. To subculture, cells were dissociated using 0.25% trypsin plus 1 mM EDTA in balanced salt solution (Gibco) and reseeded at $1.87X10^5$ cells per 175 cm^2 cell culture flasks.

Cell solution preparation

Solutions for cell incubation experiments were prepared by dissolving the individual compounds in F-12K supplemented with 10% FBS and 1X PSA (solutions used as freezing buffers did not contain PSA) and sterile filtered prior to use.

Cryopreservation of A549 cell monolayers

Cells to be frozen in the monolayer format were seeded at $0.4X10^6$ cells per well in 500 µL of cell culture medium in 24-well plates (Corning Incorporated, Corning, NY). Plates had a total available volume of 3.4 mL with an approximate growth area of 1.9 cm^2, no coverslips were used and plates were used with the accompanying lid. Cells were allowed to attach to the entire free surface of the bottom of the well and formed a confluent layer not greater in height than one cell. Before experimental treatments, cells were allowed to attach for 2 h to the plates in a humidified atmosphere of 5% CO_2 and 95% air at 37 °C. The medium was exchanged against medium that was or was not supplemented with solutes as indicated in the figure. Control cells received no additional solutes and experimental cells were incubated with 23.1 mg/mL L-proline for 24 h in a humidified atmosphere of 5% CO_2 and 95% air at 37 °C. Following the incubation period, the culture medium was removed and cells were exposed for 10 min at room temperature to different concentrations of solutes dissolved in F-12K supplemented with 10% FBS and 10% DMSO. After 10 min, the freezing solutions were removed and the plates placed inside a CoolCell® MP plate (BioCision, LLC, Larkspur, CA), transferred to a -80 °C freezer and frozen at a rate of 1 °C/min. After 24 h at -80 °C, cells were rapidly thawed by addition of 500 µL cell culture medium warmed to 37 °C. Cells were placed in a humidified atmosphere

for 24 h and then dissociated using 0.25% trypsin plus 1 mM EDTA in balanced salt solution. The number of viable cells was then determined by counting with a haemocytometer (Sigma Aldrich Co Ltd) at room temperature after 1:1 dilution of the sample with 0.4% trypan blue solution (Sigma Aldrich Co Ltd). The initial cell medium was discarded such that any non-attached cells were not included in the assessment. The percentage of recovered cells was calculated by dividing the number of cells with intact membranes after freezing and thawing by the number of cells present prior to freezing (i.e. after application of pre-treatments), multiplied by 100.

Cytotoxicity Measurements

Cells were seeded at $6 \cdot 10^4$ cells per well in 200 μL of cell culture medium with indicated concentrations of polyproline in 96-well plates (ThermoFisher). Cells were incubated for 24 h in a humidified atmosphere of 5% CO_2 and 95% air at 37 °C. Following the incubation period, resazurin sodium salt (Sigma Aldrich) was dissolved in phosphate buffered saline (Sigma Aldrich) and added to wells in an amount of 1/10th initial well volume. Absorbance was measured at 570/600 nm every 60 minutes until control cells reached ~70% reduction, and the viability reported relative to the control cells.

Haemolysis assay

Samples containing 250 μL ovine red blood cells (RBCs) and 250 μL of PPro solution (at indicated concentration) were incubated at 37 °C for 1 hour. After centrifugation, 10 μL of the supernatant was added to 90 μL of PBS buffer in a 96 well plate. The absorbance was measured at 450 nm and compared against a PBS buffer and deionised water (to lyse cells) controls to determine the % haemolysis relative to the controls.

Statistical analyses

Data were analysed with a one-way analysis of variance (ANOVA) on ranks followed by comparison of experimental groups with the appropriate control group (Holm–Sidak method) followed by Tukey's post hoc test. Excel 2013 (Microsoft, Redmond, WA) and R (R Foundation for Statistical Computing, Vienna, Austria) were used for the analyses. Data sets are presented as mean ± (SEM).

Confocal Microscopy sample preparation and measurements

The solution of 20 mg/mL of poly(proline) in PBS was prepared as previously reported. This solution was stained with 60 µL/mL of 1 mM solution of Sulphorhodamine B in deionized water. The fluorophore absorbs the 552 nm wavelength laser light and emits in a wide band: The fluorescent light was integrated in the 575-625 nm range. Water at the liquid/solid phase transition expels all ions, such that the ice crystals will be constituted by pure water. In this way, we can highlight the ice crystals that upon freezing will grow in the solution as black bodies in the fluorescent liquid. The experimental setup for a similar experiment has been described elsewhere.[3] A thin Hale-Shaw cell was built as follows: on a squared thin glass slide (20 x 20 mm^2, thickness $\lambda \approx 170$ µm, VWR) a 10 µl drop was deposited by using a pre-calibrated pipette. The sessile drop was carefully covered with a circular thin glass slide (Ø=15 mm, $\lambda \approx 170$ µm, VWR). The circular contour of such Hale-Shaw cell was sealed with nail polish (L'Oreal). With time, the solvent of the seal eventually evaporates, making the seal porous. The sample was therefore rapidly mounted on the cryostage and frozen only once, while the seal was still effective. The sample was discarded after the experiment.

Confocal Microscopy Measurement

To simulate – to a first approximation – the protocol of splat experiments reported elsewhere, [4] the sample was brought as quickly as possible (a few minutes) at the minimum temperature T_{min} ≈ –22°C that the cryostage can sustain. Nevertheless, this protocol is largely unable to provide the same crystallization feature of the splat experiments owing to the ice crystallization in a fraction of a second being more feasible with the latter. At a temperature below –15°C ice crystals burst from the cold side towards the hot side. The rapid imaging rate of the microscope is able to capture the growing ice crystal and the flow of fluorescent liquid.

Additional Data

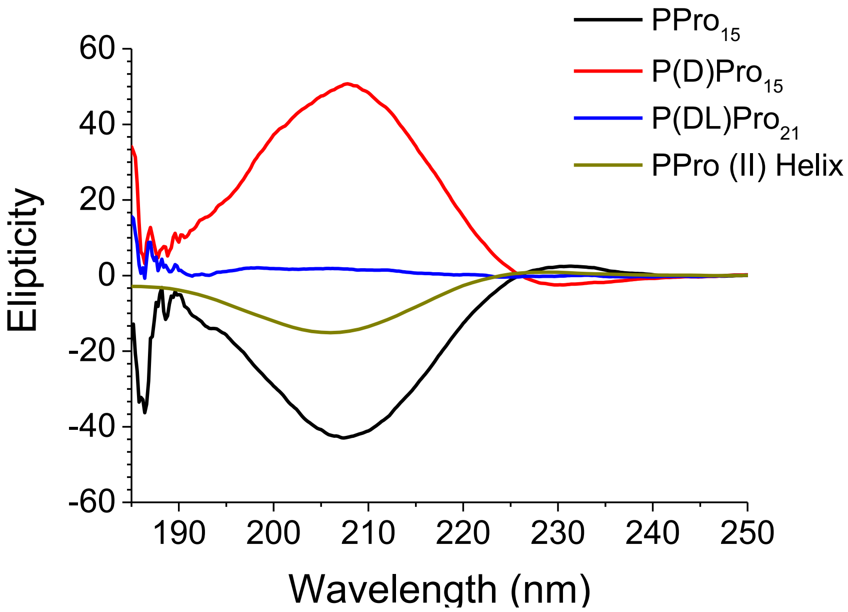

Figure S1. Circular dichroism spectra. Synthesised proline polypeptides compared to a polyproline II helical reference (PPro (II) Helix) [2] not corrected for concentration to enable comparison against reference standard

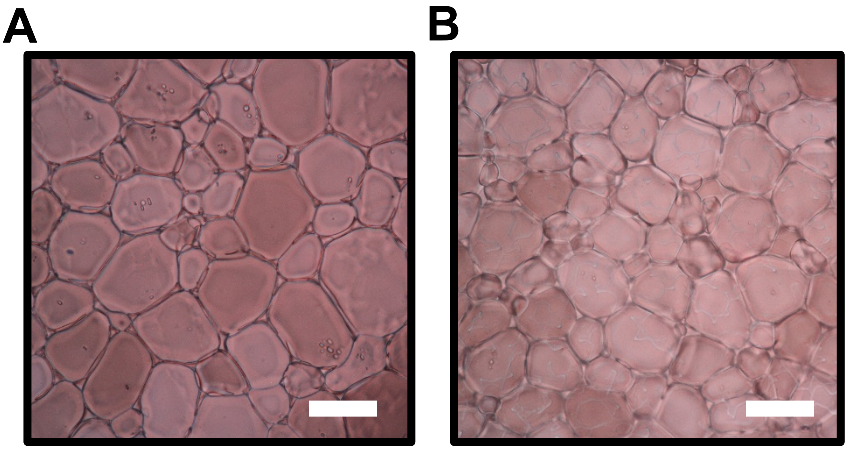

Figure S2. SPLAT assays. A) PBS control; B) L-proline, 20 mg.mL^{-1}. Scale bar = 100 υm.

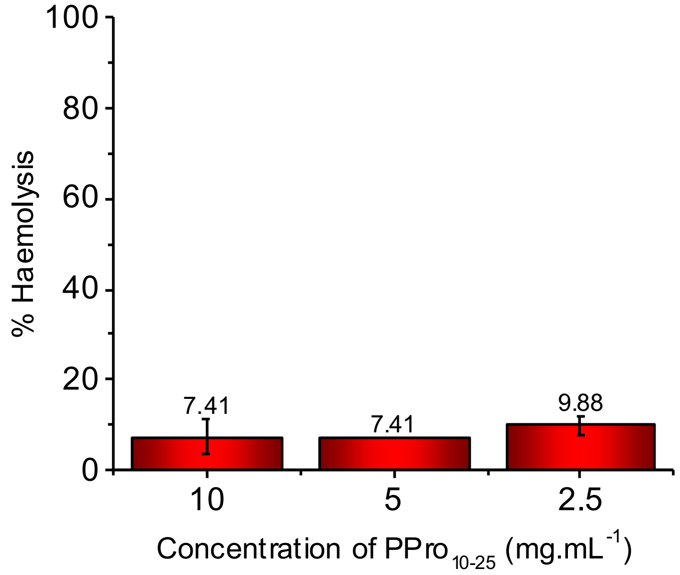

Figure S3. Heamolysis (1 hour at 37 ºC) using $PPro_{10\text{-}25}$. Values reported relative to a positive control for 100 % lysis. Errors bars represent the standard deviation from n = 3.

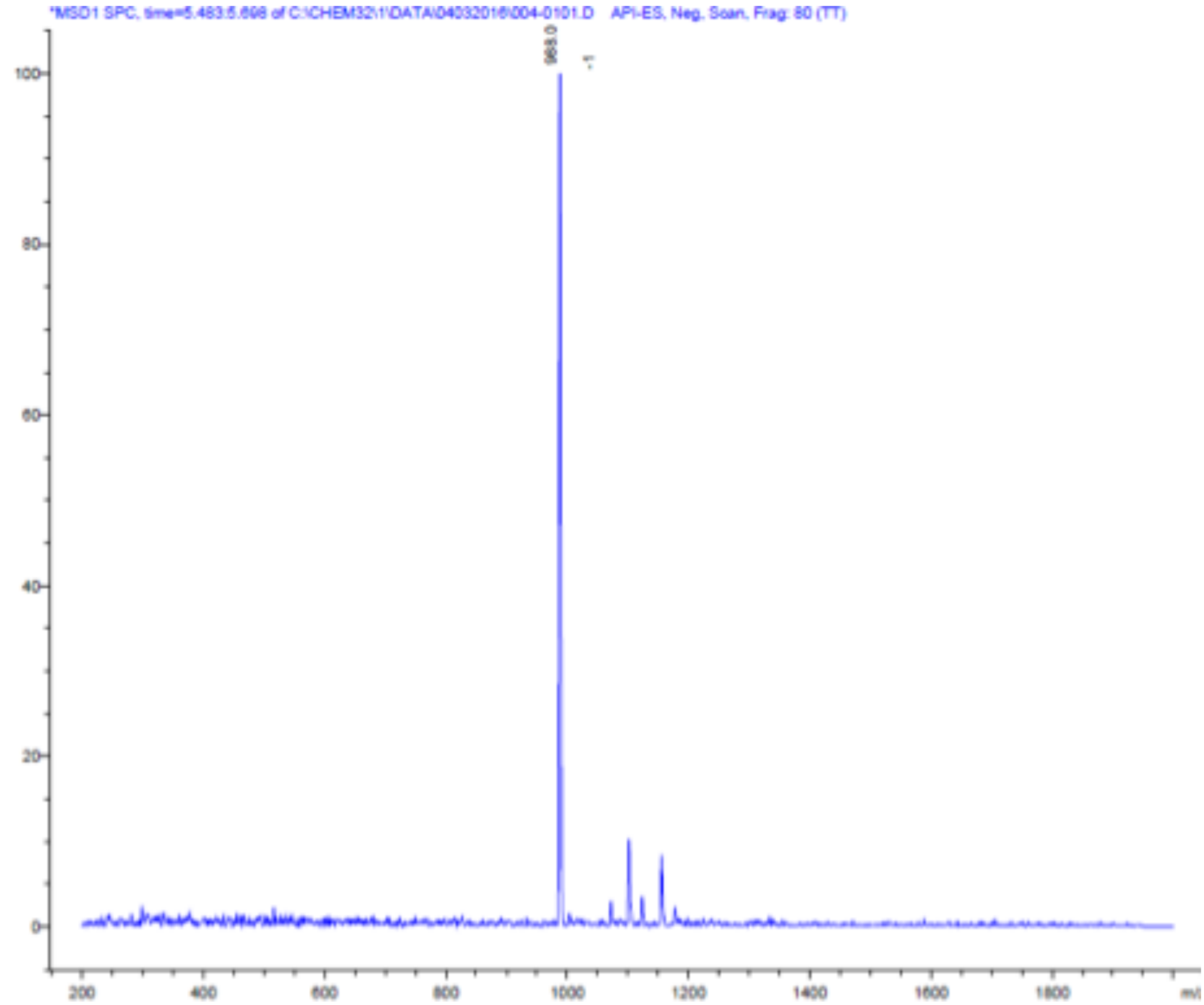

Figure S4. $PPro_{10}$ ESI Mass Spec (Provided by Supplier)

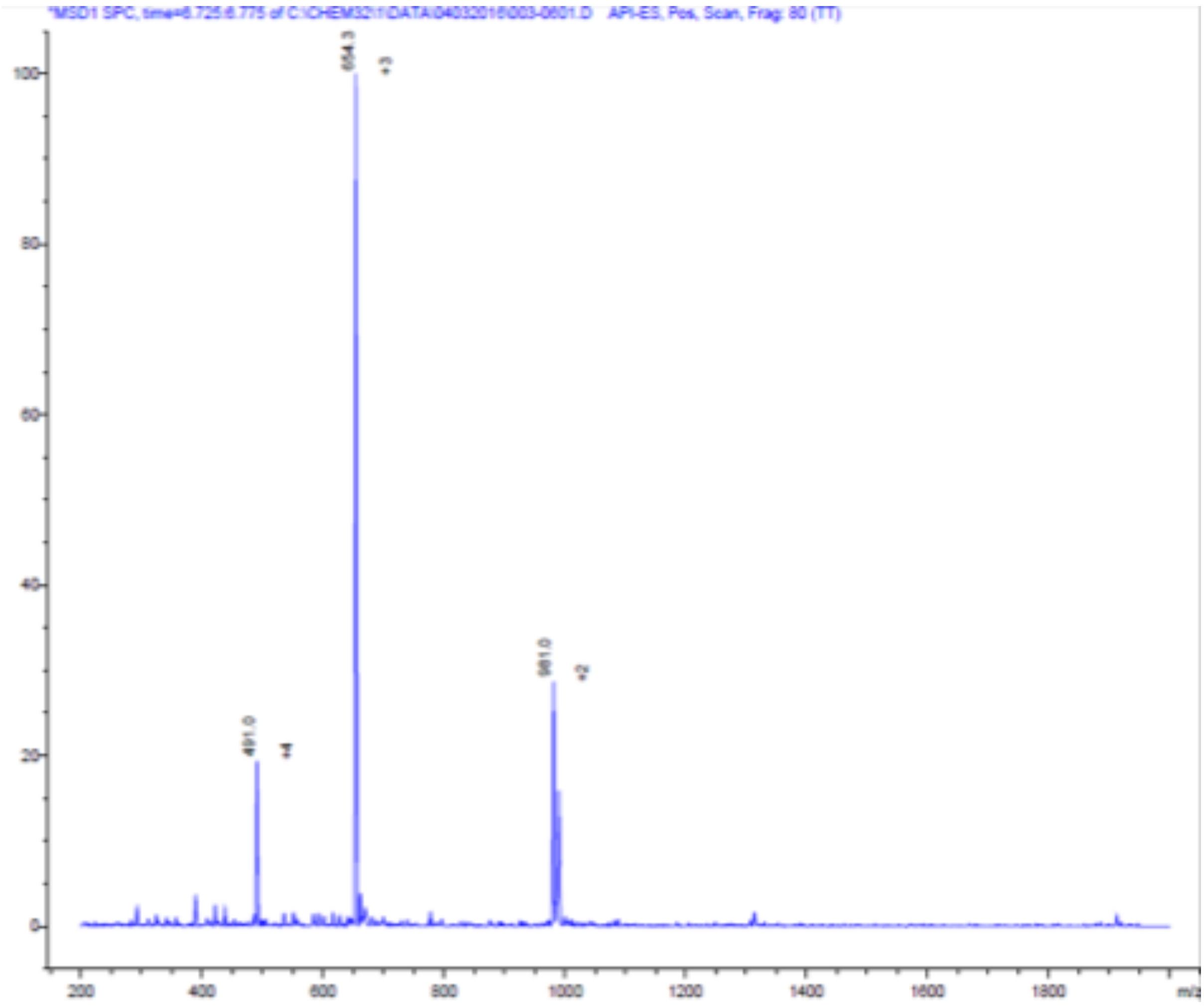

Figure S5. $PPro_{20}$ ESI Mass Spec (Provided by Supplier)

Cytotoxicity of PPro upon extended exposure to A549 Cells.

The cryopreservation method introduced in this work only requires exposure of the IRI active polymer (in this case PPro) for 10 minutes (see experimental, above). After which time the media is removed, and the monolayer frozen, with minimal liquid (which in turn reduces the opportunities for unwanted ice growth). The cells are also thawed by addition of warm media, hence diluting the PPro. Considering this, the cells are only exposed to solutions of poly(proline) for short periods of time, and essentially at dilute conditions. To evaluate the cytotoxicity of polyproline, A549 cells were exposed to PPro (including concentration above that which is optimal for cryopreservation) for 24 hours, and the cell viability determined (Figrue S5). Not that 5 mg.mL-1 PPro lead to a reduction in viability, which indicates some cytotoxicity. It should be noted this is still better than what would be expected for DMSO solution and was only observed for these very long exposure times. These results will guide the development of this cryoprotecant in terms of optimising exposure times.

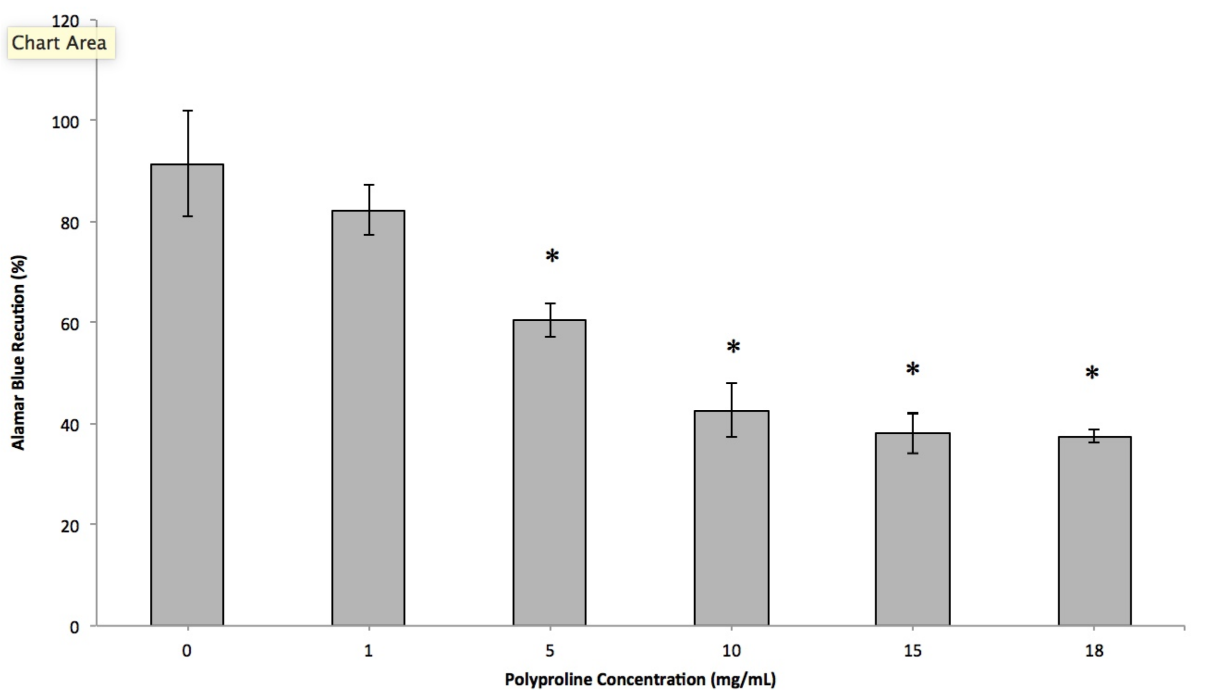

Figure S6. A549 Cell recovery after 24 hours exposure to PPRo as determined by alamar blue (resazurin) reduction. Error bars ± S.E.M from n= 3; * $P < 0.05$ compared to control (0 mg.m^{L-1} proline).

Data points for Figure 1

CD DATA (Figure 1A)

λ (nm)	[θ] PPro$_{15}$	λ (nm)	[θ] P(D)Pro$_{15}$	λ (nm)	[θ] P(DL)Pro$_{14\text{-}24}$
260	550394.31	260	442165.5	260	246849.8
259.8	582274.75	259.8	510005.7	259.8	264495.4
259.6	596721.98	259.6	534083.9	259.6	303294.1
259.4	566510.99	259.4	519574.4	259.4	297284.2
259.2	505907.36	259.2	497586.1	259.2	300964.2
259	454927.72	259	455692.4	259	277777.9
258.8	410229.71	258.8	414608.3	258.8	266765.5
258.6	341749.39	258.6	355809.1	258.6	254649.1
258.4	294305.87	258.4	312592	258.4	240835.3
258.2	262742.02	258.2	275324.3	258.2	201215.5
258	227744.12	258	233517.1	258	149104.9
257.8	170125.432	257.8	189089	257.8	70944.19
257.6	135429.59	257.6	180836.9	257.6	24049.49
257.4	146414.917	257.4	206591.4	257.4	17041.87
257.2	122097.345	257.2	193249.7	257.2	-5892.44
257	141808.619	257	185244.9	257	-9680.93
256.8	128960.774	256.8	136464.3	256.8	-67076.1
256.6	103983.207	256.6	103988.4	256.6	-90315.3
256.4	81794.746	256.4	62922.87	256.4	-127594
256.2	33956.786	256.2	50261.17	256.2	-158901
256	20625.925	256	85558.88	256	-151209
255.8	-1639.2356	255.8	108190.9	255.8	-159768
255.6	0	255.6	100738.8	255.6	-134455
255.4	27955.243	255.4	104845.1	255.4	-114280
255.2	48301.254	255.2	90398.9	255.2	-139461
255	44177.972	255	47142.15	255	-162404
254.8	77085.34	254.8	25633.41	254.8	-128463
254.6	149172.191	254.6	44960.62	254.6	-83827.4
254.4	190383.04	254.4	79531.04	254.4	-38265.6
254.2	151891.232	254.2	49685.77	254.2	-32756.8
254	117403.855	254	-2164.66	254	-45794.4
253.8	118977.117	253.8	-12560.1	253.8	-21327.3
253.6	106699.653	253.6	29511.38	253.6	16661.18
253.4	82690.886	253.4	42571.49	253.4	-8130.57
253.2	53692.972	253.2	45821.13	253.2	-47112.5
253	40658.979	253	82936.2	253	-48168
252.8	60361.084	252.8	81673.13	252.8	-49672.6
252.6	42497.796	252.6	12590.32	252.6	-57747.7
252.4	48354.019	252.4	0	252.4	-54393.9

252.2	52651.512	252.2	8467.018	252.2	-88725.5
252	57247.43	252	19634.64	252	-109362
251.8	104930.901	251.8	88325.32	251.8	-75669.8
251.6	100547.6	251.6	121333	251.6	-97280.3
251.4	73496.455	251.4	146685.3	251.4	-62972.2
251.2	45569.065	251.2	144041.7	251.2	-51501.4
251	88148.517	251	187288.1	251	0
250.8	156837.994	250.8	285069.4	250.8	118206.7
250.6	149092.957	250.6	340199.3	250.6	170051.4
250.4	87623.289	250.4	321998	250.4	120445
250.2	56966.305	250.2	332580.4	250.2	111415.7
250	59937.407	250	360014.7	250	151522.9
249.8	84503.061	249.8	351100	249.8	189975.6
249.6	28765.748	249.6	277396.9	249.6	120500.9
249.4	-25167.521	249.4	254481.3	249.4	70335.61
249.2	-41124.868	249.2	277227.3	249.2	93306.86
249	-42493.644	249	290166	249	135765.8
248.8	-51087.419	248.8	290015.5	248.8	155311.4
248.6	-95344.452	248.6	287382.4	248.6	155712.8
248.4	-157497.99	248.4	221704.7	248.4	107692.9
248.2	-172823.19	248.2	173467.1	248.2	67066.62
248	-176830.22	248	101054.3	248	-17248.9
247.8	-198612.65	247.8	43082.36	247.8	-56845
247.6	-262209.18	247.6	-66126.5	247.6	-134735
247.4	-298743.32	247.4	-160436	247.4	-210730
247.2	-245082.18	247.2	-194064	247.2	-230189
247	-204965.21	247	-206929	247	-223253
246.8	-228166.24	246.8	-324574	246.8	-303276
246.6	-238049.73	246.6	-394431	246.6	-388498
246.4	-214855.62	246.4	-421596	246.4	-435197
246.2	-176171.09	246.2	-407873	246.2	-396674
246	-134956.78	246	-397147	246	-347719
245.8	-132472.67	245.8	-420594	245.8	-376158
245.6	-166716.12	245.6	-466126	245.6	-458632
245.4	-224375.81	245.4	-536364	245.4	-531684
245.2	-248872.61	245.2	-591264	245.2	-580718
245	-242390.3	245	-602877	245	-585566
244.8	-261010.29	244.8	-629187	244.8	-620908
244.6	-233053.49	244.6	-593655	244.6	-587096
244.4	-188462.74	244.4	-578574	244.4	-591500
244.2	-143811.96	244.2	-564407	244.2	-587875
244	-142914.44	244	-548977	244	-571003
243.8	-148699.56	243.8	-537988	243.8	-539456
243.6	-155345.18	243.6	-522924	243.6	-481024

243.4	-156152.91	243.4	-514000	243.4	-411760
243.2	-195474.43	243.2	-550368	243.2	-390002
243	-219611.39	243	-611583	243	-388564
242.8	-210669.02	242.8	-690189	242.8	-430933
242.6	-151656.13	242.6	-718030	242.6	-447520
242.4	-94874.584	242.4	-748538	242.4	-462033
242.2	-60675.079	242.2	-785934	242.2	-491448
242	-8431.3799	242	-831279	242	-520803
241.8	57767.468	241.8	-862729	241.8	-485509
241.6	63318.865	241.6	-877280	241.6	-495648
241.4	77563.339	241.4	-890931	241.4	-480916
241.2	84351.167	241.2	-923867	241.2	-452392
241	101002.763	241	-998890	241	-491229
240.8	86150.194	240.8	-1069519	240.8	-575948
240.6	114431.369	240.6	-1171070	240.6	-656496
240.4	162689.027	240.4	-1237981	240.4	-726409
240.2	277509.3	240.2	-1282525	240.2	-738351
240	420602.79	240	-1297453	240	-732557
239.8	526669.09	239.8	-1297360	239.8	-753659
239.6	580105.33	239.6	-1308146	239.6	-744075
239.4	633915.25	239.4	-1352443	239.4	-734993
239.2	725414.95	239.2	-1394774	239.2	-721731
239	790796.84	239	-1447522	239	-681803
238.8	876360.91	238.8	-1486584	238.8	-610225
238.6	905919.69	238.6	-1538437	238.6	-608456
238.4	934603.09	238.4	-1591719	238.4	-624712
238.2	967746.43	238.2	-1711492	238.2	-650820
238	1016082.63	238	-1815514	238	-707071
237.8	1051180.87	237.8	-1909868	237.8	-763308
237.6	1062417.22	237.6	-1994517	237.6	-784617
237.4	1136893.72	237.4	-2099078	237.4	-783056
237.2	1186191.8	237.2	-2217739	237.2	-799087
237	1254096.03	237	-2293219	237	-761592
236.8	1350553.91	236.8	-2374460	236.8	-766208
236.6	1451551.31	236.6	-2472637	236.6	-776464
236.4	1603320.75	236.4	-2535505	236.4	-698202
236.2	1729972.32	236.2	-2599913	236.2	-683371
236	1837830.9	236	-2657903	236	-659511
235.8	1924227.1	235.8	-2748451	235.8	-618893
235.6	1993634.7	235.6	-2873098	235.6	-655502
235.4	2109440.9	235.4	-2970410	235.4	-670547
235.2	2341693.4	235.2	-3030891	235.2	-604797
235	2541491.1	235	-3078051	235	-522217
234.8	2701377.7	234.8	-3160226	234.8	-490454

234.6	2881661	234.6	-3165416	234.6	-373161
234.4	3068795.1	234.4	-3197853	234.4	-274220
234.2	3263627.7	234.2	-3199254	234.2	-119587
234	3456021	234	-3157648	234	18089.52
233.8	3595130.3	233.8	-3149327	233.8	81351.46
233.6	3637930.5	233.6	-3248421	233.6	68245.14
233.4	3686301.3	233.4	-3323884	233.4	121713
233.2	3748858.1	233.2	-3385299	233.2	166836.9
233	3831777	233	-3482006	233	190507.2
232.8	3933241.5	232.8	-3581515	232.8	193263.7
232.6	3992822.7	232.6	-3683430	232.6	153810.2
232.4	4030225.3	232.4	-3760190	232.4	67833.44
232.2	4074340.3	232.2	-3793388	232.2	43980.37
232	4154664.2	232	-3863298	232	48639.71
231.8	4183537.9	231.8	-3916097	231.8	31770.59
231.6	4247288.4	231.6	-3908139	231.6	56946.16
231.4	4295884.1	231.4	-3937584	231.4	31518.74
231.2	4282649.6	231.2	-4016974	231.2	-24384.1
231	4238292.4	231	-4100290	231	-76701.1
230.8	4146048.8	230.8	-4216702	230.8	-209060
230.6	4111673.7	230.6	-4291542	230.6	-264700
230.4	4138333	230.4	-4277944	230.4	-261945
230.2	4148574.6	230.2	-4233206	230.2	-279096
230	4054947	230	-4260800	230	-357963
229.8	3891046.8	229.8	-4277339	229.8	-411633
229.6	3768441.7	229.6	-4282529	229.6	-502315
229.4	3673949.1	229.4	-4189437	229.4	-476121
229.2	3675748.3	229.2	-4038149	229.2	-432697
229	3682097.4	229	-3926771	229	-430620
228.8	3570720	228.8	-3764445	228.8	-428012
228.6	3369278.8	228.6	-3628658	228.6	-431365
228.4	3152354.1	228.4	-3578609	228.4	-475141
228.2	2920914.7	228.2	-3486573	228.2	-545825
228	2718089.5	228	-3286862	228	-581452
227.8	2621244.1	227.8	-3004110	227.8	-553302
227.6	2478968.9	227.6	-2686967	227.6	-544778
227.4	2332853.1	227.4	-2385618	227.4	-479269
227.2	2072349.7	227.2	-2094234	227.2	-455630
227	1783491.6	227	-1789477	227	-463533
226.8	1510307.3	226.8	-1455740	226.8	-472130
226.6	1230651.07	226.6	-1122832	226.6	-497874
226.4	937758.61	226.4	-646928	226.4	-542110
226.2	670210.65	226.2	-183136	226.2	-593612
226	289069.16	226	212054.8	226	-733799

225.8	-206387.27	225.8	624076.7	225.8	-865223
225.6	-671861.07	225.6	1149182	225.6	-835330
225.4	-1150782.2	225.4	1688378	225.4	-848137
225.2	-1607804.9	225.2	2370429	225.2	-847000
225	-2123678.8	225	3027638	225	-887761
224.8	-2661034.1	224.8	3731991	224.8	-838976
224.6	-3273938.5	224.6	4370516	224.6	-830047
224.4	-4003790.9	224.4	4946416	224.4	-931240
224.2	-4643562.2	224.2	5633140	224.2	-948610
224	-5284440.7	224	6327562	224	-934876
223.8	-5930318.9	223.8	7063140	223.8	-889481
223.6	-6674859	223.6	7878420	223.6	-856053
223.4	-7435297.8	223.4	8700654	223.4	-838458
223.2	-8100932.6	223.2	9596068	223.2	-676662
223	-8756775.6	223	10497069	223	-474108
222.8	-9523390.5	222.8	11516887	222.8	-283033
222.6	-10370952	222.6	12568554	222.6	-85208.8
222.4	-11147982	222.4	13643749	222.4	134360
222.2	-11933350	222.2	14646907	222.2	234579.3
222	-12795374	222	15554966	222	258338.3
221.8	-13619546	221.8	16476780	221.8	296962.2
221.6	-14422578	221.6	17520056	221.6	399167.3
221.4	-15262856	221.4	18541621	221.4	474545.2
221.2	-16155519	221.2	19675982	221.2	507642.2
221	-16987441	221	20793389	221	538940.6
220.8	-17994595	220.8	21975671	220.8	558014.5
220.6	-18962876	220.6	23262272	220.6	580584.4
220.4	-19912646	220.4	24491956	220.4	578351.1
220.2	-20932308	220.2	25770080	220.2	578551.2
220	-21907163	220	27083669	220	565657.4
219.8	-23038064	219.8	28364215	219.8	447639.8
219.6	-24170522	219.6	29542518	219.6	381873.6
219.4	-25254367	219.4	30737775	219.4	417111.9
219.2	-26314857	219.2	31891166	219.2	433881.2
219	-27449391	219	33139707	219	393898
218.8	-28559532	218.8	34550176	218.8	509197
218.6	-29722784	218.6	36057179	218.6	624399.4
218.4	-30944510	218.4	37383743	218.4	696246.8
218.2	-32143919	218.2	38630208	218.2	680583.8
218	-33295926	218	39993448	218	683509.4
217.8	-34402953	217.8	41476750	217.8	809066.4
217.6	-35609628	217.6	42917840	217.6	949587.2
217.4	-36739491	217.4	44245788	217.4	1035929
217.2	-37993049	217.2	45533427	217.2	1015648

217	-39301448	217	46851168	217	1011393
216.8	-40532170	216.8	48014247	216.8	1044032
216.6	-41717566	216.6	49209158	216.6	1145471
216.4	-42837914	216.4	50521536	216.4	1259006
216.2	-43979887	216.2	51576317	216.2	1265665
216	-45109750	216	52810326	216	1248012
215.8	-46112458	215.8	54056791	215.8	1328618
215.6	-47095790	215.6	55275057	215.6	1385543
215.4	-48175483	215.4	56378278	215.4	1418718
215.2	-49306211	215.2	57637545	215.2	1410022
215	-50441610	215	59119809	215	1499911
214.8	-51572338	214.8	60429419	214.8	1561863
214.6	-52631098	214.6	61696990	214.6	1704008
214.4	-53583636	214.4	62823566	214.4	1880512
214.2	-54473721	214.2	64132830	214.2	1955262
214	-55397195	214	65285183	214	2044679
213.8	-56318593	213.8	66429059	213.8	2188054
213.6	-57199336	213.6	67567918	213.6	2305106
213.4	-58131806	213.4	68634463	213.4	2526113
213.2	-59084690	213.2	69795466	213.2	2681639
213	-60048819	213	71025150	213	2742888
212.8	-60965719	212.8	72383892	212.8	2863799
212.6	-62019808	212.6	73478290	212.6	3003777
212.4	-63087737	212.4	74456086	212.4	3149505
212.2	-64141307	212.2	75387172	212.2	3299902
212	-64866004	212	76351647	212	3386037
211.8	-65551776	211.8	77114577	211.8	3361519
211.6	-66176825	211.6	77645341	211.6	3339278
211.4	-66733712	211.4	78308277	211.4	3371708
211.2	-67460312	211.2	78908241	211.2	3309033
211	-68106467	211	79600068	211	3261239
210.8	-68794142	210.8	80420780	210.8	3292864
210.6	-69409157	210.6	81240627	210.6	3296751
210.4	-70002201	210.4	82042136	210.4	3336495
210.2	-70640744	210.2	82885511	210.2	3353929
210	-71085008	210	83668163	210	3321890
209.8	-71372707	209.8	84240966	209.8	3374537
209.6	-71701580	209.6	84597000	209.6	3445170
209.4	-72111763	209.4	84934004	209.4	3435510
209.2	-72340296	209.2	85502309	209.2	3425344
209	-72558103	209	86114902	209	3377366
208.8	-72855490	208.8	86765555	208.8	3367223
208.6	-73208583	208.6	86867279	208.6	3521208
208.4	-73499915	208.4	86891845	208.4	3759419

208.2	-73622226	208.2	87351852	208.2	3822324
208	-73855430	208	87562912	208	3858825
207.8	-74000231	207.8	87768090	207.8	3975665
207.6	-74182227	207.6	87629517	207.6	4017571
207.4	-74349172	207.4	87372439	207.4	4037834
207.2	-74259558	207.2	86866414	207.2	4070057
207	-73966496	207	86536849	207	4155364
206.8	-73576727	206.8	86154692	206.8	4236508
206.6	-73416010	206.6	85677212	206.6	4262107
206.4	-73482096	206.4	85089185	206.4	4216107
206.2	-73284530	206.2	84829339	206.2	4296791
206	-73042157	206	84463098	206	4280921
205.8	-72832308	205.8	83933372	205.8	4336972
205.6	-72338047	205.6	83338771	205.6	4283497
205.4	-71728395	205.4	82704726	205.4	4209460
205.2	-71037606	205.2	82309767	205.2	4076704
205	-70477605	205	82153375	205	4120289
204.8	-70238346	204.8	81540090	204.8	4162701
204.6	-69873143	204.6	81033892	204.6	4147015
204.4	-69310374	204.4	80265080	204.4	4145980
204.2	-68689477	204.2	79500593	204.2	4109525
204	-68002148	204	78703236	204	4095610
203.8	-67376926	203.8	78358620	203.8	4054900
203.6	-66829554	203.6	77789277	203.6	3967684
203.4	-66087384	203.4	77058179	203.4	3932402
203.2	-65305251	203.2	76510807	203.2	3976355
203	-64354097	203	76016027	203	3936335
202.8	-63317481	202.8	75370737	202.8	3860527
202.6	-62211319	202.6	74632027	202.6	3771954
202.4	-61161555	202.4	73922208	202.4	3826372
202.2	-60453639	202.2	73051153	202.2	3848130
202	-59644172	202	72230960	202	3932448
201.8	-58940062	201.8	71313195	201.8	3828419
201.6	-58118139	201.6	70271562	201.6	3776669
201.4	-57000213	201.4	69267989	201.4	3899558
201.2	-55855299	201.2	68421327	201.2	3951009
201	-54789619	201	68104910	201	4025529
200.8	-53836043	200.8	67587640	200.8	4096760
200.6	-52934194	200.6	67034040	200.6	4183999
200.4	-52223510	200.4	66103819	200.4	4145773
200.2	-51377367	200.2	65294006	200.2	4169739
200	-50567035	200	64401326	200	4244857
199.8	-49579032	199.8	63141886	199.8	4210725
199.6	-48742404	199.6	61949051	199.6	4272135

199.4	-47929131	199.4	60805694	199.4	4373542
199.2	-47071743	199.2	59560786	199.2	4449304
199	-46066440	199	58212943	199	4506896
198.8	-45122725	198.8	57159546	198.8	4548273
198.6	-44217243	198.6	56199396	198.6	4616169
198.4	-43333559	198.4	55186481	198.4	4724039
198.2	-42458179	198.2	53851613	198.2	4714471
198	-41455125	198	52721750	198	4573228
197.8	-40477502	197.8	51269934	197.8	4597171
197.6	-39390716	197.6	49406032	197.6	4629049
197.4	-38408768	197.4	47968229	197.4	4492107
197.2	-37293610	197.2	46758440	197.2	4445210
197	-36148177	197	45379457	197	4433848
196.8	-35092704	196.8	43982309	196.8	4269536
196.6	-34298980	196.6	42813002	196.6	3942108
196.4	-33460622	196.4	41612036	196.4	3829684
196.2	-32673645	196.2	40283223	196.2	3513204
196	-31824734	196	39214083	196	3297441
195.8	-31013018	195.8	38322614	195.8	3106748
195.6	-30120511	195.6	37291707	195.6	2986435
195.4	-29128010	195.4	36259762	195.4	2875851
195.2	-28196405	195.2	35369331	195.2	2746085
195	-27199752	195	34762966	195	2490923
194.8	-26541660	194.8	34106950	194.8	2396830
194.6	-26154140	194.6	33225688	194.6	2326266
194.4	-25638254	194.4	32670012	194.4	2099599
194.2	-24999192	194.2	32196511	194.2	2020060
194	-24380371	194	31162663	194	1958997
193.8	-24254254	193.8	29665694	193.8	1420620
193.6	-24230726	193.6	28812631	193.6	905298.4
193.4	-24299407	193.4	27991573	193.4	413155.9
193.2	-24171560	193.2	27315662	193.2	134043.5
193	-23562081	193	26821747	193	203461
192.8	-23093597	192.8	26637848	192.8	267333.6
192.6	-22214238	192.6	26088573	192.6	647585.7
192.4	-21106865	192.4	25628566	192.4	931240.1
192.2	-20454309	192.2	25317339	192.2	511835.1
192	-19835661	192	23880401	192	414269.1
191.8	-19184489	191.8	22406787	191.8	440719.1
191.6	-18764964	191.6	21418438	191.6	255047
191.4	-18193026	191.4	20897016	191.4	-381377
191.2	-17090964	191.2	20517454	191.2	44319.16
191	-15484209	191	18808906	191	393484
190.8	-14692613	190.8	17749800	190.8	791308.1

190.6	-14041061	190.6	17319030	190.6	1502015
190.4	-11112032	190.4	18516363	190.4	2240844
190.2	-8905849.7	190.2	18620163	190.2	3204797
190	-8738714.4	190	15273443	190	3073145
189.8	-8898082	189.8	16586392	189.8	5868266
189.6	-7323937.7	189.6	19336383	189.6	6663882
189.4	-9986528.8	189.4	17514174	189.4	4003081
189.2	-9373935.8	189.2	12649985	189.2	2348277
189	-12986107	189	11203030	189	2322149
188.8	-18413082	188.8	8979340	188.8	4183309
188.6	-20081321	188.6	13082018	188.6	6261980
188.4	-16418721	188.4	13285206	188.4	5085001
188.2	-5518232.9	188.2	12184719	188.2	9972432
188	-8390742.2	188	11304045	188	6027288
187.8	-14113928	187.8	8158991	187.8	2956719
187.6	-12283190	187.6	10165722	187.6	11381067
187.4	-20430781	187.4	13814396	187.4	9119339
187.2	-28004548	187.2	18274855	187.2	12090111
187	-28706063	187	22038816	187	20206650
186.8	-48261464	186.8	19144872	186.8	20142917
186.6	-58922243	186.6	15093126	186.6	9812076
186.4	-62774953	186.4	5522022	186.4	-1637798
186.2	-56822715	186.2	9386582	186.2	4718197
186	-55715169	186	10862359	186	1362350
185.8	-57923860	185.8	24323281	185.8	6731709
185.6	-43837335	185.6	42044882	185.6	19081145
185.4	-35808578	185.4	54249340	185.4	25842110
185.2	-22204377	185.2	56859045	185.2	34812800
185	-26073695	185	59040229	185	35990630
184.8	-36469092	184.8	53041454	184.8	28445250
184.6	-31906390	184.6	56200088	184.6	40191580
184.4	-24378641	184.4	60140163	184.4	46489670
184.2	-35957358	184.2	61401852	184.2	37349010
184	-33385540	184	55510510	184	36446720
183.8	-30890707	183.8	51395186	183.8	29684720
183.6	-27235736	183.6	48053864	183.6	8749108
183.4	-18466539	183.4	53925830	183.4	8162286
183.2	-12400104	183.2	64554258	183.2	23405490
183	-22487751	183	59962665	183	17420775
182.8	-22976649	182.8	60747393	182.8	15840790
182.6	-12764528	182.6	62327748	182.6	22918442
182.4	-17044202	182.4	47164125	182.4	16274317
182.2	-29108980	182.2	32040638	182.2	2135495
182	-44019677	182	26467270	182	-1.2E+07

181.8	-52380594	181.8	32163987	181.8	-1.6E+07
181.6	-49937661	181.6	41231609	181.6	-1258620
181.4	-47278478	181.4	47707345	181.4	12820683
181.2	-40097767	181.2	34361606	181.2	-4362847
181	-30717015	181	32062782	181	-4984077
180.8	-9468757.1	180.8	44062408	180.8	17100385
180.6	4882976.9	180.6	52803579	180.6	23922990
180.4	6672696.5	180.4	63743234	180.4	38637700
180.2	5679382.4	180.2	66613304	180.2	53022590
180	18432631	180	66226822	180	65201780

IRI Data (Figure 1B)

$PPro_{10-1000}$ 5 mg/ml	
Avg Set 1	99.266
Avg Set 2	99.448
Avg Set 3	128.455
Cum Average	109.0563
**STD DEV**	**19.13652**
**% MLGS**	**79.02633**

$PPro_{10-1000}$ 10 mg/ml	
Avg Set 1	103.716
Avg Set 2	100.377
Avg Set 3	108.021
Cum Average	104.038
**STD DEV**	**8.717811**
**% MLGS**	**75.38986**

$PPro_{10-1000}$ 20 mg/ml	
Avg Set 1	72.712
Avg Set 2	74.805
Avg Set 3	94.545
Cum Average	80.68733
**STD DEV**	**17.58934**
**% MLGS**	**58.46908**

$PPro_{10}$ 5 mg/ml	
Avg Set 1	100.582
Avg Set 2	171.522
Avg Set 3	116.791
Cum Average	129.6317
**STD DEV**	**37.05471**
**% MLGS**	**93.93599**

$PPro_{10}$ 10 mg/ml	
Avg Set 1	89.368
Avg Set 2	91.748
Avg Set 3	90.759
Cum Average	90.625
**STD DEV**	**13.5148**
**% MLGS**	**65.67029**

$PPro_{10}$ 20 mg/ml	
Avg Set 1	97.569
Avg Set 2	77.741
Avg Set 3	-
Cum Average	87.655
**STD DEV**	**13.7729**
**% MLGS**	**63.51812**

$PPro_{20}$ 5 mg/ml	
Avg Set 1	105.192
Avg Set 2	113.484
Avg Set 3	105.151
Cum Average	107.9423
**STD DEV**	**10.63947**
**% MLGS**	**78.21908**

$PPro_{20}$ 10 mg/ml	
Avg Set 1	86.676
Avg Set 2	147.657
Avg Set 3	149.398
Cum Average	127.9103
**STD DEV**	**40.75635**
**% MLGS**	**92.68865**

$PPro_{20}$ 20 mg/ml	
Avg Set 1	96.27
Avg Set 2	75.218
Avg Set 3	101.992
Cum Average	91.16
**STD DEV**	**13.22746**
**% MLGS**	**66.05797**

PPro$_{15}$ 5 mg/ml	
Avg Set 1	91.391
Avg Set 2	95.493
Avg Set 3	95.687
Cum Average	94.19033
**STD DEV**	**14.51387**
**% MLGS**	**68.25386**

PPro$_{15}$ 10 mg/ml	
Avg Set 1	73.662
Avg Set 2	89.935
Avg Set 3	102.55
Cum Average	88.71567
**STD DEV**	**19.05693**
**% MLGS**	**64.28671**

PPro$_{15}$ 20 mg/ml	
Avg Set 1	46.842
Avg Set 2a	75.218
Avg Set 2b	71.791
Cum Average	64.617
**STD DEV**	**15.28502**
**% MLGS**	**46.82391**

P(D)Pro$_{15}$ 5 mg/ml	
Avg Set 1	82.376
Avg Set 2	78.778
Avg Set 3	75.119
Cum Average	78.75767
**STD DEV**	**10.18674**
**% MLGS**	**57.07077**

P(D)Pro$_{15}$ 10 mg/ml	
Avg Set 1	64.522
Avg Set 2	88.988
Avg Set 3	88.458
Cum Average	80.656
**STD DEV**	**16.25138**
**% MLGS**	**58.44638**

P(D)Pro$_{15}$ 20 mg/ml	
Avg Set 1	61.31
Avg Set 2	67.368
Avg Set 3	80.032
Cum Average	69.57
**STD DEV**	**13.22802**
**% MLGS**	**50.41304**

P(DL)Pro$_{14-24}$ 5 mg/ml	
Avg Set 1a	90.729
Avg Set 2	90.254
Avg Set 1b	91.995
Cum Average	90.99267
**STD DEV**	**9.706077**
**% MLGS**	**65.93671**

P(DL)Pro$_{14-24}$ 10 mg/ml	
Avg Set 1	84.264
Avg Set 2	93.582
Avg Set 3	81.885
Cum Average	86.577
**STD DEV**	**10.91247**
**% MLGS**	**62.73696**

P(DL)Pro$_{14-24}$ 20 mg/ml	
Avg Set 1	68.397
Avg Set 2	66.639
Avg Set 3	79.321
Cum Average	71.45233
**STD DEV**	**12.31481**
**% MLGS**	**51.77705**

References.

1 C. A. Knight, J. Jallett, A. L. DeVries, *Cryobiology*, 1988, **25**, 55-60

2 Protein Circular Dichroism Data Bank, 2016, CD0004553000

3 D. E. Mitchell, N. R. Cameron, and M. I. Gibson., *Chem. Commun.*, 2015, **51**, 12977 – 12980.

4 M. Marcellini, C. Noirjean, D. Dedovets, J. Maria, and S. Deville, *ACS Omega*, 2016, **1**, 1019 – 1026.
